# Days of Flooding Associated with Increased Risk of Influenza

**DOI:** 10.1155/2022/8777594

**Published:** 2022-06-03

**Authors:** Eric Kontowicz, Grant Brown, James Torner, Margaret Carrel, Kelly K. Baker, Christine A. Petersen

**Affiliations:** ^1^Department of Epidemiology, College of Public Health, University of Iowa, Iowa City 52242, IA, USA; ^2^Center for Emerging Infectious Diseases, University of Iowa Research Park, Coralville 52241, IA, USA; ^3^Department of Biostatistics, College of Public Health, University of Iowa, Iowa City 52242, IA, USA; ^4^Department of Geographical and Sustainability Sciences, College of Liberal Arts and Sciences, University of Iowa, Iowa City 52242, IA, USA; ^5^Department of Occupational and Environmental Health, College of Public Health, University of Iowa, Iowa City 52242, IA, USA; ^6^Immunology Program, Carver College of Medicine, University of Iowa, Iowa City 52242, IA, USA

## Abstract

Influenza typically causes mild infection but can lead to severe outcomes for those with compromised lung health. Flooding, a seasonal problem in Iowa, can expose many Iowans to molds and allergens shown to alter lung inflammation, leading to asthma attacks and decreased viral clearance. Based on this, the hypothesis for this research was that there would be geographically specific positive associations in locations with flooding with influenza diagnosis. An ecological study was performed using influenza diagnoses and positive influenza polymerase chain reaction tests from a de-identified large private insurance database and Iowa State Hygienic Lab. After adjustment for multiple confounding factors, Poisson regression analysis resulted in a consistent 1% associated increase in influenza diagnoses per day above flood stage (95% confidence interval: 1.00–1.04). This relationship remained after removal of the 2009–2010 influenza pandemic year. There was no associated risk between flooding and influenza-like illness as a nonspecific diagnosis. Associated risks between flooding and increased influenza diagnoses were geographically specific, with the greatest risk in the most densely populated areas. This study indicates that populations who live, work, or volunteer in flooded environments should consider preventative measures to avoid environmental exposures to mitigate illness from influenza in the following year.

## 1. Introduction

Influenza viruses, members of the Orthomyxoviridae family [1], are among the leading causes of human respiratory infections globally [2]. The estimated burden of influenza in the United States falls between nine and 49 million cases, causing 1,700 to 59,000 deaths annually [3]. A recent report from the Iowa Department of Public Health indicated 1,889 influenza-associated hospitalizations and 270 influenza-related deaths in Iowa [4]. Influenza typically causes mild infection but can lead to hospitalization or death for individuals that are younger than five, older than 65, or those who have concomitant lung disease (i.e., asthma, chronic obstructive pulmonary disease, etc.) [5–8]. Environmental and occupational factors also influence influenza prevalence. Those who work or live in close proximity to large animal production facilities, as can be common in Iowa, are at an increased risk of lung disease [9, 10]. Poor air quality around and in animal production facilities posed greater risk for lung disease compared with areas where animal production facilities are not present [11, 12].

Geographic spread and evolution of influenza viruses is driven by complex relationships among viral evolution, climatic factors, and transmission differences at the local, regional, and global scale [13–16]. In temperate regions, increased influenza transmission has been shown experimentally and epidemiologically to occur in cooler, less humid settings with seasonal peaks in winter months [17–21]. Compartmental modeling has demonstrated an interaction between viral transmission and specific humidity that was sufficient to explain observed differences in epidemic intensity in several US cities [22]. In addition, changes in specific humidity have been found to correlate with intense epidemics [22], and decreases in both indoor and outdoor absolute humidity have been associated with increased influenza virus airborne survivability and transmission [23, 24]. Anti-influenza vaccination, the most prevalent prevention strategy for influenza, reduced influenza transmission at both local and regional scales [25–27] At regional and local scales, influenza burden and timing are closely correlated with human contact patterns, population density, timing of school sessions, and level of susceptibility [28–32].

Water-saturated areas caused by flooding are prone to mold growth and growth of other spore-forming microbes that affect respiratory health [33]. One study found via passive air samples and quantitative PCR that flooded homes had significant differences in bacterial and fungal community composition and significant increases in fungus concentrations compared with nonflooded homes [34]. Additionally, it has been suggested that homes impacted by floods never return to a baseline fungal concentration [34, 35]. A recent meta-analysis indicated that residential dampness and mold increased the odds of respiratory infections by 45% [36]. Increases in flooding-induced allergens led to increased asthma attacks and subsequently increased risk of influenza infection even months later [37–39]. Flooding affects many members of a population [40, 41], exposure to these postflood environments could influence influenza rates at a population level months after initial flooding events.

Iowa is an ideal state to evaluate a flooding and influenza relationship due to the large number of floods that have occurred each year after large precipitation events [42], which is projected to rise [43–45]. It is critical to understand to what extent flooding and subsequent water-saturated areas increase the risk of influenza diagnosis in Iowans. We hypothesized there would be a positive association between flooding and influenza diagnoses for the Iowa population as exposure to flooded environments can alter susceptibility to influenza. It is further hypothesized that flooding in Iowa would be spatially associated with geographically specific increased influenza diagnoses.

## 2. Methods

### 2.1. Influenza Outcome Data

Influenza diagnoses were quantified from two sources: a database of de-identified fully insured administrative claims data from a private insurance company and polymerase chain reaction (PCR) test results from Iowa State Hygienic Lab provided by Dr. Lucy Desjardin. International Classification of Disease (ICD)-9 and ICD-10 codes were used to ensure complete case inclusion for influenza diagnosis between 2007 and 2017 (Supplemental Table 1). Influenza diagnoses and positive PCR tests were summed for each three-digit ZCTA based on county of diagnoses or testing (here on referred to as influenza diagnoses). Similar data were collected for influenza-like illness (ILI) diagnoses (Supplemental Table 2).

**Figure 1:**
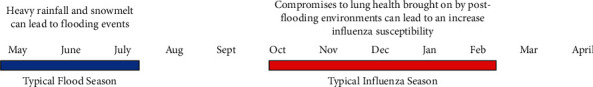
Flood-influenza (flu) year. All data for this study were aggregated to this yearly structure for each three-digit ZCTA in Iowa.

To temporally assess how flooding impacts the following influenza season, a hypothesized flood-flu year was defined as May 1^st^ to April 31^st^ of the following year (Figure 1). Aggregation was performed at this temporal level to ensure a given flood season only influences the following influenza season.

### 2.2. Population-Level Covariates

Multiple variables were used to account for known population-level factors that can influence influenza rates. Yearly asthma diagnosis and asthma attack rates were calculated for each three-digit ZCTA of Iowa using diagnosis codes from the aforementioned de-identified insurance claims database (Supplemental Table 3) [25–27, 37–39]. Yearly vaccination rates were calculated using National Drug Code (NPC), Current Procedural Terminology (CPT) codes, and ICD-9 and ICD-10 codes to ensure complete capture of all of vaccinated against influenza (Supplemental Table 4).

Topologically Integrated Geographic Encoding and Referencing (TIGER) shape files from the 2010 US census were used to calculate the population counts and population density for each three-digit ZCTA. Total population for each three-digit ZCTA served as the denominator when calculating population percent working in animal production and vaccination, asthma, and asthma attack rates. Data from the 2017 agricultural census was used to determine the total number of individuals per three-digit ZCTA whose primary vocation was animal production.

### 2.3. Environmental-Level Covariates

**Figure 2:**
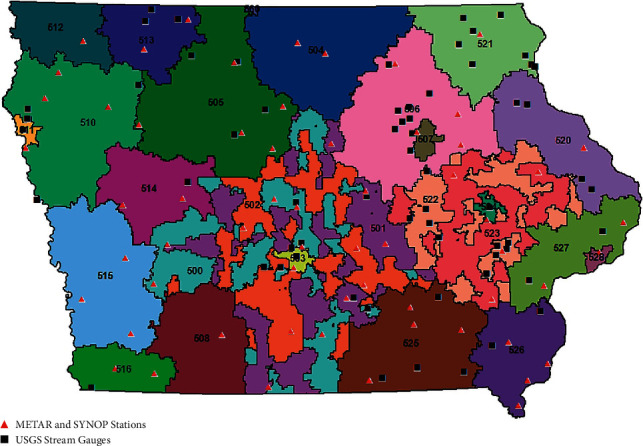
Spatial distribution of USGS stream gauges and METAR and SYNOP weather stations in Iowa. Locations of all SYNOP and METAR weather stations and USGS stream gauges used in the study. Each unique three-digit ZCTA is shown as a different color. Map created using ArcMap 10.7.1 (Esri, Redlands, CA).

Daily records from meteorological aerodrome report (METAR) stations, surface synoptic observations (SYNOP) stations, and United States Geology Survey (USGS) stream gauges were collected for the three-digit ZCTA regions across Iowa (Figure 2). Monthly averages of temperature and relative and absolute humidity were taken by averaging the daily data for each weather station. Weather stations were geotagged in ArcMap to locate them via three-digit ZCTA (determined by US Census 2010 TIGER shape files) based on latitude and longitude. For each three-digit ZCTA, seasonal averages for temperature and absolute and relative humidity were calculated per flood-flu index year by averaging monthly weather station data into two seasons: flood (May, June, and July) and influenza (October, November, December, January, February, and March).

Stream height data were collected, and flood-stage-level indicators were identified from 63 USGS stream gauges across Iowa (Figure 2). Average, daily stream height was calculated for each stream gauge and dichotomized based on the flood stage indication. Flooding was considered present when the daily average stream height was greater than the flood stage level for each stream gauge. Based on these daily dichotomized flood indications, monthly days above flood stage was calculated for every stream gauge. These data were aggregated for the three-digit ZCTA level by averaging days above flood stage for all stream gages in each three-digit ZCTA.

### 2.4. Statistical Model

Biological relevance, bivariate correlation, and multicollinearity of covariates were used to determine inclusion into final models. Pearson correlations were performed for each covariate and influenza diagnoses data. Collinearity of covariates was measured using variance inflation factors (VIFs). If a variable had a VIF greater than five, it was considered for removal from the final modeling procedure depending on biological relevance and Pearson correlation significance to ensure final model convergence.

Poisson regression with a conditional autoregressive spatial component (three-digit ZCTA level) was implemented using a Bayesian approach via R2OpenBugs to determine the relationship between days above flood stage (flooding) and influenza diagnoses [46]. These models were run at the three-digit ZCTA level and aggregated to flood-flu year for each spatial unit. Model outcomes were total influenza (or ILI) diagnoses per three-digit ZCTA for each flood-flu year (2007–2017). Offset was total population per three-digit ZCTA. The conditional autoregressive component provided accounting of adjoining geographic locations that were likely to impact neighboring areas. All priors used for modeling had noninformative distributions; mean 0 and precision 0.001. Each model used three chains and 2,500,000 iterations using Markov Chain Monte Carlo (MCMC) sampling. Convergence of MCMC output was assessed via Gelman–Rubin diagnostics. Models were considered convergent when the Gelman–Rubin diagnostic was less than 1.05 for all model variables [47, 48].

## 3. Results

The combination of influenza diagnoses from de-identified insurance claims and Iowa State Hygienic Lab influenza PCR test results was used to quantify influenza diagnoses for the Iowa population for each flood-flu year. The largest rate of influenza diagnoses was during the 2009–2010 pandemic influenza season (Table 1) [49].

To establish flooding frequency in each Iowa ZCTA, stream height data were collected from 63 USGS stream gauges. Known environmental factors that influence risk of influenza diagnosis [17–21] were addressed by collecting temperature and humidity data from 60 METAR and five SYNOP weather stations across Iowa (Figure 2). An average of 45 (±5.60) USGS stream gauge stations reported stream height data each year. Average days above flood stage averaged 17 to 22 days each year (Table 1).

### 3.1. Multiple Environmental and Population-Level Factors Associated with Influenza Diagnoses

The relationship between flooding and influenza diagnoses is complicated by a variety of intersecting environmental and population determinants. To drive informed modeling choices, bivariate analyses were conducted to evaluate associations between aggregated covariates and influenza diagnoses (Table 2). Ten of fourteen suspected and literature-based environmental- and population-level determinants were significantly correlated with influenza diagnoses (Table 2).

Collinearity was calculated via variance inflation factors (VIFs) among the above fourteen influenza risk factors (correlation matrix of covariates provided in Supplemental Table 5). All humidity and temperature values, except relative humidity during influenza season, had VIFs greater than 10. Every humidity and temperature value was excluded from modeling, except for absolute humidity during the influenza season, which had the highest level of correlation to the outcome of influenza infection (−0.27, *p* < 0.005) (Table 2). Absolute humidity during the influenza season was selected to remain in the final models for biological relevance as a better measure compared with relative humidity when assessing influenza risk [3, 22, 23, 28]. Temperature and humidity are directly related with high collinearity [50]. Many of the population-level covariates had VIF values between five and ten (Table 2). Percent of the population in animal production and asthma attack rate were included in the multivariable model due to their biological relevance for influenza transmission and environmental exposures.

The list of final model variables included: (influenza) vaccination rate, asthma attack rate, flooding, average absolute humidity during the influenza season (flu), population density, and percent of the population in animal production. VIFs for each variable when modeling standardized influenza diagnoses as an outcome using only these specified environmental exposure variables were all less than two and a half (range 1.09–2.32).

### 3.2. Increased Influenza Diagnoses Are Associated with Increased Flooding

A Bayesian conditional autoregressive (CAR) Poison model was fit to estimate the association between flooding exposures and influenza diagnoses. This model indicated that flooding exposures (mean: 1.01 [95% CI: 1.00–1.02], posterior probability of risk >1: 0.768), asthma attack rates (1.04 [95% CI: 0.989–1.09], 0.914), and percent of the population in animal production (1.01 [95% CI: 0.890–1.15], 0.536) had the greatest estimated risk associated with influenza diagnoses. Asthma attack rates (0.914) and the total average days above flood stage (0.768) had the greatest probability of the estimated risk being greater than one for an associated increase in influenza diagnoses (Table 3).

These results can be interpreted as a four percent increase in influenza diagnoses per each increase of asthma attacks or a one percent increase in influenza diagnoses per day above flood stage across Iowa.

**Figure 3:**
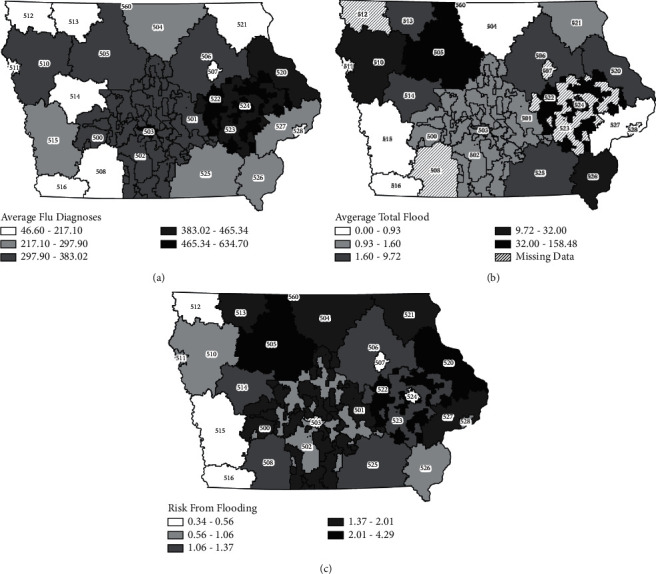
Estimated risk of increased influenza diagnoses from days above flood stage. (a) Average influenza diagnoses. Average influenza diagnoses and positive PCR tests per each three-digit ZCTA from 2007 to 2017. (b) Average total days above flood stage. Average days above flood stage per each three-digit ZCTA from 2007–2017. Dashed areas indicate missing data. (c) Risk of influenza diagnosis from flooding. Risk calculations for each three-digit ZCTA were calculated from the multivariate Poisson regression model. Dark shading indicates a high associated risk, while light shading indicates a low associated risk. Three-digit ZCTA boundaries were taken from TIGER shape files from the US Census Bureau. Maps created using ArcMap 10.7.1 (Esri, Redlands, CA).

The greatest concentration of influenza diagnoses were in densely populated areas, in particular the Des Moines (ZCTA 503) and Cedar Rapids (524) metro areas, and toward the eastern section of the state (Figure 3(a)), consistent with the role of population density as a risk factor for influenza transmission [32]. Flooding predominantly occurred in the northwest section of the state in the region of forks of the Des Moines River and within the Iowa City/Cedar Rapids corridor, with the Iowa and Cedar rivers, and their tributaries (Figure 3(b)). The associated risk for an increase in influenza diagnoses due to flooding is concentrated along the north-central portion of the state as well as the eastern border (Figure 3(c)). It was found that ZCTA 503 had the highest risk of an increase of influenza diagnoses associated with flooding (risk = 4.293). ZCTA 524 was found to have the lowest risk of an increase of influenza diagnoses associated with flooding (risk = 0.437). Maps of other covariates used in the final modeling are provided in Supplemental Figure 1.

It is well understood that when there is a major reassortment of different influenza strains to make a novel strain, there is limited preexisting immunity to this strain, greatly increasing the population susceptibility and changing the seasonal dynamics of transmission [51]. To determine if the relationship between flooding and influenza rates was biased by the novel pandemic strain from the 2009–2010 influenza season, an analysis was performed omitting this year. Similar associations were found between influenza diagnoses and flooding with the 2009–2010 pandemic year omitted (Table 4).

Unlike the previous analysis (0.718 [95% CI: 0.371–1.47]), average absolute humidity was found to have the highest associated risk (1.40 [95% CI: 0.467–4.97], 0.727). Percent of the population working in animal production (1.04 [95% CI: 0.844–1.24], 0.712) was found to have the highest associated risk of the population-level factors, which is a 3% increase from the previous analysis. Both asthma attack rates (1.03 [95% CI: 0.980–1.08], 0.841) and the total average days above flood stage (1.01 [95% CI: 1.00–1.02], 0.755) maintained levels of risk greater than one. Similar to the original influenza regression analysis, asthma attack rates had the greatest probability of the estimated risk being greater than one (0.841), followed by total average days above flood stage (0.755).

### 3.3. Flooding Has No Associated Risk with Influenza-Like Illness Diagnoses

To determine if the relationship between flooding and influenza diagnoses is specific to influenza or is broadly related to seasonal infectious respiratory diseases, a separate analysis was performed to assess the association of flooding with influenza-like illness (ILI). This analysis found no association between total average days above flood stage and ILI (1.00 [95% CI: 0997–1.00], 0.007) (Table 5).

Furthermore, the probability of the estimated risk between total days above flood stage and ILI being greater than one was close to zero (0.007). Like previously presented analyses, both asthma attack rates (1.02 [95% CI: 0.993–1.04], 0.948) and percent of the population working in animal production (1.05 [95% CI: 0.933–1.09], 0.946) had associated risks greater than one. The probability for the estimated risk being greater than one for asthma attack rates increased by 0.054, and for percent of the population working in animal production increased by 0.41.

### 3.4. Sensitivity Analyses to account for Missing Flooding Data

All results presented above used complete data only. Flooding data were missing for four three-digit ZCTAs of Iowa across the study period. Two separate sensitivity analyses were performed to determine the impacts that the missing flood data could have on the estimated risk assessments between flooding and influenza diagnoses. The first analysis implied a best-case scenario in which all the missing flood data were set to zero. This implied spatial units without flooding data did not experience flooding (Supplemental Table 6). Results from this analysis were similar to the original analysis results except average absolute humidity (1.56 [95% CI: 0.889–2.77], 0.941) had greater risk associated with influenza diagnosis and the risk for percent population in animal production increased by 10%. It was found that the probability of the estimated risk being greater than one for percent of the population in animal production increased from the original analysis by 0.453 (0.536 to 0.989). The associated one percent risk for an increase in influenza diagnoses per one day above flood stage remained the same, while the probability of the estimated risk being greater than one decreased (0.680 from 0.768 previously).

It is more likely that these areas with missing data would experience similar flooding to areas around it. Missing flood data were imputed based on neighboring three-digit ZCTAs. This analysis produced similar trends as the above analyses (Supplemental Table 7). The average absolute humidity had the largest risk associated with it (1.58 [95% CI: 0.903–2.77], 0.947). Compared with the missing values set to zero analysis, this is a 0.02 increase in risk and a smaller 95% confidence interval. The percent of the population working in the animal production industry (1.11 [95% CI: 1.02–1.20], 0.992) was found to have the second highest associated risk. This is the same compared with the missing set to zero analysis, however, with a higher value for the 97.5% quantile. The total average days above flood stage (1.01 [95% CI: 0.999–1.02], 0.740) was the only other factor that had an associated risk, which remained the same compared with the missing set to zero analysis. It was also found that the probability of risk being greater than one for an increase in influenza diagnoses remains high (0.740) but decreased slightly compared with the original analysis (0.768).

## 4. Discussion

Influenza viruses are estimated to infect one billion individuals each year [52], and their spread are driven by climate factors, viral evolution, and population susceptibility [13–16]. Flooding is both a disaster and a common event in Iowa that can cause an increase in respiratory health problems with long-lasting impacts that influences population susceptibility to influenza [33, 42, 53, 54]. We hypothesized that those who are exposed to postflooding environments, through living circumstances, volunteer clean-up efforts, or work hazards, may be at greater risk for future influenza infection. Risk can come from being exposed to mold in postflooded environments or exposure to increased levels of allergens in the air following floods. These exposures can lead to both acute and chronic complications, most notably potential fibrosis due to asthma attacks or even micro foreign bodies, that can persist for months after the initial exposure [33, 53–57]. This study demonstrates a positive association between flooding and an increase in influenza rates for Iowans.

All results in this study found a similar 1% estimated risk of increase in influenza diagnoses per day above flood stage for the state of Iowa; this relationship persisted when omitting the pandemic influenza year of 2009–2010 (Tables 3-4). This suggests that the flooding and influenza relationship is not driven by pandemic strains of the influenza virus and could persist for seasonal influenza as well. If there was widespread flooding across the state, as seen during major flooding events in 1994 and 2010, the impact across Iowa, 3.155 million people, would be 31,555 diagnoses for each day above flood stage. Two other known factors that can influence lung health, asthma attack rates [5–8] and percent of population in animal industry [9, 10], also consistently showed associated risks (Tables 3–5). These results suggest when adjusting for other known risk factors of influenza, asthma attack or humidity for example, flooding still has a direct level of estimated risk. The state of Iowa experiences approximately 20 days of flooding each year. This implies, on average, that Iowans are at 20% increased risk of influenza each year compared with other populations that do not experience flooding.

The associated risk between flooding and influenza diagnoses was heterogeneous at the three-digit ZCTA spatial scale of Iowa, and it was found that the northeast and eastern three-digit ZCTAs had consistently higher levels of risk (Figure 1). These ZCTAs experienced a larger burden of flooding compared with the other Iowa ZCTAs. Further, many of the animal feed operations are densely located in the northeast region of the state. Of note, ZCTA 503, the Des Moines regional area, has a very large risk (risk = 4.29). This ZCTA has the highest population density (1830.56 individuals per mi^2^ compared with state average 228.27 individuals per mi^2^), and this was in the highest flu diagnoses category, suggesting that despite having lower average days above flood stage, the floods that did occur could have had a greater impact on more individuals. A significant percentage of the population living in this area has been shown to be living in poverty (12%) or as asset-limited/income-constrained households (17%) [58]. This analysis did not consider socioeconomic status of residents within each three-digit ZCTA as it was outside the limits of the model. These factors together may describe why such a large risk could be attributed to flooding in the capital region of Iowa. The three-digit ZCTA 524 had a rather low associated risk attributed to flooding (risk = 0.437) despite having a large average influenza diagnoses and moderate rates of flooding. ZCTA 524 has the second highest average rate of influenza vaccination (759.58 vaccinations per 100,000) for the whole state (531.85 vaccinations per 100,000), and thus the risk from flooding may be mitigated by the high vaccination rates. These findings suggest that in areas where large amounts of the population could be exposed to flooded environments, there is a large, estimated risk for an associated increase in influenza diagnoses. These findings highlight the need for those who work, volunteer, or live in postflooding environments to take preventative measures during clean-up efforts such as wearing N95 or filtered masks and preventative measures, like vaccination, to avoid influenza infection in subsequent influenza seasons.

Unlike the association between flooding exposure and influenza diagnoses, there was no estimated association between influenza-like illness (ILI) diagnoses and flooding (1.00 [0.997–1.00], 0.007). ILI is a very broad respiratory illness diagnosis, which can be caused by a myriad of respiratory alignments [59]. The lack of an association between flooding and ILI diagnoses suggests evidence toward a specific relationship between flooding exposures and influenza. This relationship may be due to how populations respond to influenza rather than flooding exposures specifically altering transmission of the influenza virus. This relationship may not be generalizable to other microbe-specific diagnoses, such as SARS-CoV-2. Further studies need to be performed to access the generalizability of these findings.

All analyses and results presented here are at the population level may not apply equally to every individual residing within each three-digit ZCTA. Further, studies performed at a different spatial scale may find different results. More granular studies need to be conducted to provide greater understanding of the risk at an individual level, including a more nuanced measure of flood exposure and potential corresponding respiratory risk factors.

There are many strengths to this study. First, unlike prior studies that have only evaluated health outcomes immediately following single flooding events [60–63], this study analyzed over ten years of flooding exposure and influenza records. This helps better account for overall changes that have occurred due to climate and influenza strain differences. Daily averages of stream height were used to determine the existence of flooding, and thus small-scale or flash floods (floods typically lasting less than a couple hours) were not captured in these data. It is unlikely that flash floods would create conditions of a postflooded environment evaluated in this study as they are not as likely to cause as much property damage [64]. Analyses used in this study also incorporated an autoregressive component during modeling to allow for neighboring ZCTA influenza diagnoses to influence each other since disease transmission does not uphold to arbitrary postal codes.

This study also has limitations. Data collection and analysis for this study were limited to administrative boundaries and in some areas noncontiguous spatial units were present (ZTCAs 501, 502, 522, and 523). Areas with noncontiguous spatial units can create issues when evaluating associations between exposure (i.e., flooding) and outcome (i.e., influenza diagnoses) [65]. For this research, associations could be biased in these areas as individuals may be exposed to flooding environments in different ZCTAs compared with where they were diagnosed with influenza. Aggregating to different spatial units, such as counties or grid squares, may affect findings and interpretation of these current results [65, 66]. Flooding data were aggregated, which assumes that everyone within each spatial unit experiences flooding in the exact same way. Currently, there is not an alternative measure to account for individual experiences of flooding at the state level. Similarly, we used a standard definition for a flooded state that may not precisely reflect the conditions that can influence influenza susceptibility. For example, water levels directly beneath flood stage levels may still create for local damp humid environments that can influence mold growth or similar settings that can influence population susceptibility for the upcoming influenza season. In addition, the presence of *a priori* uncertainty concerning the true generating model and the important covariates required the use of model selection, rather than a purely confirmatory statistical analysis. Further study is needed, particularly at the individual level to characterize the exposure and determine individual-level influenza risk associated with flooding.

## 5. Conclusions

These results found a 1% increase in estimated risk of influenza diagnoses associated with each day of flooding. Additionally, these results found that the risk associated with flooding is geographically specific with excess risk affecting more densely populated areas. These findings suggest that in areas where large amounts of the population could be exposed to flooded environments, there is a large, estimated risk for an associated increase in influenza diagnoses. While additional research needs to be performed to determine the risk at the individual level, in the meantime those who work, volunteer, or live in postflooding environments should be advised to take preventative measures to avoid influenza infection in subsequent influenza seasons.

## Figures and Tables

**Table 1:** Summary table of influenza diagnosis and USGS stream gauge reporting.

Date range	Physician diagnosed	PCR + tests	Average days above flood stage	Number of unique USGS stations	Stations reporting flooding
5/2007–4/2008	11,114	568	12.8	38	15
5/2008–4/2009	5045	507	20.0	44	30
5/2009–4/2010	11,534	1880	20.8	45	23
5/2010–4/2011	6567	1070	27.4	45	36
5/2011–4/2012	1693	1346	18.4	45	12
5/2012–4/2013	8604	2016	19.3	43	17
5/2013–4/2014	3972	681	21.8	44	23
5/2014–4/2015	9871	890	21.3	44	21
5/2015–4/2016	3302	385	17.9	44	19
5/2016–4/2017	575	1052	18.7	60	24

**Table 2:** Bivariate correlations between influenza diagnoses and potential covariates.

Variable (season)	Average	Standard deviation	Correlation value	Variance inflation factor
Average temperature (flood)	68.79	2.37	0.05	54.76
**Average temperature (flu)**	33.03	4.08	−0.27^*∗∗*^	25.64
Relative humidity (flood)	70.23	24.36	−0.20^*∗*^	54.73
**Relative humidity (flu)**	74.45	3.82	0.12	4.34
Absolute humidity (flood)	12.59	0.92	−0.19^*∗*^	60.85
Absolute humidity (flu)	4.25	0.55	−0.27^*∗∗*^	22.51
Flooding	19.91	39.96	0.13	1.43
**Vaccination rate**	551.13	179.55	0.40^*∗∗*^	2.24
**Asthma rate**	92.35	53.75	0.62^*∗∗*^	7.48
**Asthma attack rate**	12.21	7.30	0.54^*∗∗*^	8.00
**Population density**	224.07	456.15	0.31^*∗∗*^	3.34
**Percent older 75**	8.00	1.75	−0.33^*∗∗*^	5.21
Percent younger 5	6.51	0.57	0.12	2.17
**Percent animal production**	5.20	2.69	−0.30^*∗∗*^	6.59

Environmental factors of temperature and humidity were aggregated to flood and flu seasons before bivariate correlations were performed. All remaining variables were not aggregated to a seasonal temporal scale. Bolded covariates with ^*∗*^*p* < 0.05 and ^*∗∗*^*p* < 0.005.

**Table 3:** Results of Bayesian CAR model with influenza diagnoses as an outcome.

Variable	Mean risk	2.5% quantile	97.5% quantile	Gelman Diag	Probability of risk >1
Asthma attack rate	1.04	0.989	1.09	1.00	0.914
Vaccination rate	0.999	0.998	1.00	1.00	≤0.001
Population density	1.00	0.999	1.00	1.01	≤0.001
Percent in animal production	1.01	0.890	1.15	1.00	0.536
Average absolute humidity	0.718	0.371	1.47	1.02	0.165
Total average days above flood stage	1.01	1.00	1.02	1.00	0.768

**Table 4:** Results of Bayesian CAR model with influenza diagnoses as an outcome and 2009-2010 flu season removed.

Variable	Mean risk	2.5% quantile	97.5% quantile	Gelman Diag.	Probability of risk >1
Asthma attack rate	1.03	0.980	1.08	1.00	0.841
Vaccination rate	1.00	0.998	1.00	1.01	≤0.001
Population density	1.00	0.999	1.00	1.00	≤0.001
Percent in animal production	1.04	0.896	1.20	1.00	0.678
Average absolute humidity	1.40	0.467	4.36	1.00	0.727
Total average days above flood stage	1.01	1.00	1.02	1.00	0.755

**Table 5:** Bayesian CAR model with influenza-like illness diagnoses as an outcome.

Variable	Mean risk	2.5% quantile	97.5% quantile	Gelman diagram	Probability of risk >1
Asthma attack rate	1.02	0.993	1.04	1.00	0.948
Vaccination rate	1.00	1.00	1.00	1.01	≤0.001
Population density	1.00	1.00	1.00	1.00	≤0.001
Percent in animal production	1.05	0.993	1.09	1.00	0.946
Average absolute humidity	0.992	0.570	1.32	1.03	0.527
Total average days above flood stage	1.00	0.997	1.00	1.00	0.007

## Data Availability

Data and codes for this manuscript can be found at https://github.com/ekontowicz/Days-of-flooding-associated-with-increased-risk-of-influenza.

## Supplementary Materials

Supplementary Materials Supplemental Table 1: ICD-9 and ICD-10 codes used to capture influenza diagnoses. Supplemental Table 2: ICD-9 and ICD-10 codes used to capture influenza-like illness. Supplemental Table 3: ICD-9 and ICD-10 codes used to capture asthma and asthma attack diagnosis. Supplemental Table 4: ICD-9, ICD-10, CPT, and NDC codes used to capture influenza vaccination. Supplemental Table 5: bivariate correlation matrix of potential covariates. Supplemental Table 6: Bayesian CAR model results using imputed environmental (set to 0) data and influenza diagnosis rates as the outcomes. Supplemental Table 7: Bayesian CAR model results using imputed environmental (impute) data and influenza diagnosis rates as the outcomes. Supplemental Figure 1: maps of other covariates used in the modeling process.
