# Supplementary material for: Days of Flooding Associated with Increased Risk of Influenza
Source: J Environ Public Health. 2022 Jun 3;2022:8777594. doi: 10.1155/2022/8777594 (PMC9187473; doi:10.1155/2022/8777594)
Supplement: Supplementary Materials — Supplemental Table 1: ICD-9 and ICD-10 codes used to capture influenza diagnoses. Supplemental Table 2: ICD-9 and ICD-10 codes used to capture influenza-like illness. Supplemental Table 3: ICD-9 and ICD-10 codes used to capture asthma and asthma attack diagnosis. Supplemental Table 4: ICD-9, ICD-10, CPT, and NDC codes used to capture influenza vaccination. Supplemental Table 5: bivariate correlation matrix of potential covariates. Supplemental Table 6: Bayesian CAR model results using imputed environmental (set to 0) data and influenza diagnosis rates as the outcomes. Supplemental Table 7: Bayesian CAR model results using imputed environmental (impute) data and influenza diagnosis rates as the outcomes. Supplemental Figure 1: maps of other covariates used in the modeling process. [file 8777594.f1.pdf]

Supplemental Table 1 ICD-9 and ICD-10 codes used to capture influenza diagnoses

ICD-9 Influenza diagnosis codes	Diagnosis Description
487.0, 487.1, 487.8	Influenza with pneumonia or respiratory manifestations
488.0, 488.01, 488.8, 488.81, 488.02, 488.82, 488.09, 488.1, 488.19, 488.89, 488.11, 488.12, 487.8	Influenza due to identified avian, novel, or seasonal influenza virus
**ICD-10 Influenza diagnosis codes**	
J09, J09.X, J09.X1, J09.X2, J09.X3, J09.X9	Influenza due to certain identified influenza viruses
J10, J10.0, J10.00, J10.01, J10.08, J10.1, J10.2, J10.8, J.10,81, J10.82, J10.83, J10.89	Influenza due to other identified influenza viruses
J11, J11.0, J11.00, J11.08, J11.1, J11.2, J11.8, J11.81, J11.82, J11.83, J11.89	Influenza due to unidentified influenza virus

Supplemental Table 2 ICD-9 and ICD-10 codes used to capture influenza-like illness

ICD-9 Influenza-like Illness Codes	Diagnosis Description
079.99	Unspecified viral infection
382.9	Unspecified otitis media
460, 461.9, 465.8, 465.9	Acute nasopharyngitis, sinusitis, or upper respiratory infections, unspecified
466.0	Acute bronchitis
486	Pneumonia, organism unspecified
488.0, 488.01, 488.8, 488.81, 488.02, 488.82, 488.09, 488.1, 488.19, 488.89, 487.0, 487.1, 488.11, 488.12, 487.8	Influenza due to identified novel, avian, or seasonal influenza virus
490	Bronchitis, not specified as acute or chronic
786.2, 780.6, 780.6	Cough, fever, other physiologic disturbance of temperature regulation, and unspecified fever
**ICD-10 Influenza-like Illness Codes**	
B97.89	Other viral agents as the cause of disease classified elsewhere
H66.9, H66.90, H66.91, H66.92, H66.93	Otitis media, unspecified
J00, J01.9, J01.9, J01.90, J06.9	Acute nasopharyngitis, acute sinusitis, acute upper respiratory infection, unspecified
J09, J09.X, J09.X1, J09.X2, J09.X3, J09.X9, J10, J10.0, J10.00, J10.01, J10.08, J10.1, J10.2, J10.8, J.10,81, J10.82, J10.83, J10.89, J11, J11.0, J11.00, J11.08, J11.1, J11.2, J11.8, J11.81, J11.82, J11.83, J11.89	Influenza due to identified novel, avian, or seasonal influenza virus
J12.89, J12.9,	Other or unspecified viral pneumonia
J18, J18.1, J18.8, J18.9	Pneumonia unspecified organism

J20.9, J40	Acute bronchitis from unspecified organism, or bronchitis not specified as acute or chronic
R05, R50.9	Cough or fever, unspecified

Supplemental Table 3 ICD9 and ICD 10 Codes used to capture asthma and asthma attack diagnosis

Asthma Diagnosis ICD 9 and ICD 10 Codes
493, 493.0, 493.00, 493.01, 493.02, 493.1, 493.10, 493.11, 493.12, 493.2, 493.20, 493.21, 493.22, 493.8, 493.81, 493.82, 493.9, 493.90, 493.91, 493.92

Asthma Attack Diagnosis ICD 9 and ICD 10 Codes
493.01, 493.02, 493.11, 493.12, 493.21, 493.22, 493.91, 493.92

Supplemental Table 4 Table 1. ICD9, ICD10, CPT, and NDC codes used to capture influenza vaccination

Influenza Vaccination ICD 9 and ICD 10 codes
99.52, V0481, 3E01340, and 3E02340.

Influenza Vaccination CPT codes
90686, 90688, 90672, 90682, 90685, 90687, 90662, 90656, 90658, 90653, 90674, 90661, Q2037, 90630, 90749, 90756, 90673, 90655, 90657, 90460, 90461, 90471, 90472, 90473, 90474, 90654, 90660, Q2034, Q2035, Q2036, Q2038, and Q2039.

Influenza Vaccination NDC codes
33332-0010-01, 33332-0013-01, 33332-0014-01, 33332-0015-01, 33332-0016-01, 33332-0017-01, 33332-0018-01, 33332-0110-10, 33332-0113-10, 33332-0114-10, 33332-0115-10, 33332-0116-10, 33332-0117-10, 33332-0118-10, 33332-0316-01, 33332-0317-01, 33332-0318-01, 33332-0416-10, 33332-0417-10, 33332-0418-10, 66521-0000-01, 70461-0001-01, 70461-0002-01, 70461-0018-03, 58160-0879-52, 58160-0880-52, 58160-0881-52, 58160-0883-52, 58160-0898-52, 58160-0900-52, 58160-0901-52, 58160-0903-52, 58160-0905-52, 58160-0907-52, 42874-0012-10, 42874-0013-10, 42874-0014-10, 42874-0015-10, 42874-0016-10, 42874-0017-10, 42874-0117-10, 49281-0718-10, 62577-0613-01, 62577-0614-01, 63851-0612-01, 63851-0613-01, 70461-0200-01, 70461-0301-10, 70461-0418-10, 70461-0201-01, 70461-0318-03, 19515-0845-11, 19515-0850-52, 19515-0889-07, 19515-0890-07, 19515-0893-07, 19515-0891-11, 19515-0894-52, 19515-0895-11, 19515-0896-11, 19515-0898-11, 19515-0900-11, 19515-0901-52, 19515-0903-11, 19515-0908-52, 19515-0909-52, 19515-0912-52, 66019-0107-01, 66019-0108-10, 66019-0109-10, 66019-0110-10, 66019-0300-10, 66019-0301-10, 66019-0302-10, 66019-0303-10, 66019-0304-10, 66019-0305-10, 66521-0112-02, 66521-0112-10, 66521-0113-02, 66521-0113-10, 66521-0114-02, 66521-0114-10, 66521-0115-02, 66521-0115-10, 66521-0116-02, 66521-0116-10, 66521-0117-02, 66521-0117-10, 66521-0118-02, 66521-0118-10, 70461-0119-02, 70461-0119-10, 70461-0120-02, 70461-0120-10, 49281-0010-10, 49281-0010-25, 49281-0010-50, 49281-0011-10, 49281-0011-50, 49281-0012-10, 49281-0012-50, 49281-0013-10, 49281-0013-50, 49281-0014-50, 49281-0111-25, 49281-0112-25, 49281-0113-25, 49281-0386-15, 49281-0387-65, 49281-0388-15, 49281-0390-15, 49281-0392-15, 49281-0394-15, 49281-0396-15, 49281-0705-55, 49281-0707-55, 54868-6177-00, 54868-6180-00, 49281-0389-65, 49281-0391-65,

49281-0393-65, 49281-0395-65, 49281-0397-65, 49281-0399-65, 49281-0401-65, 49281-0403-65, 49281-0703-55, 49281-0709-55, 49281-0708-40, 49281-0710-40, 49281-0712-40, 49281-0413-10, 49281-0413-50, 49281-0414-10, 49281-0414-50, 49281-0415-10, 49281-0416-10, 49281-0416-50, 49281-0417-10, 49281-0417-50, 49281-0418-10, 49281-0418-50, 49281-0513-25, 49281-0514-25, 49281-0516-25, 49281-0517-25, 49281-0518-25, 49281-0621-15, 49281-0625-15, 49281-0627-15, 49281-0629-15, 49281-0640-15, 49281-0650-10, 49281-0650-25, 49281-0650-50, 49281-0650-70, 49281-0650-90, 66521-0200-02, 66521-0200-10, 58160-0808-15, 58160-0808-15, 66019-0200-10, 76420-0482-01, 76420-0483-01

Supplemental Table 5 Bivariate correlation matrix of potential covariates

	Avg Temp (Flood)	Avg Temp (Flu)	Relative Humidity (Flood)	Relative Humidity (Flu)	Absolute Humidity (Flood)	Absolute Humidity (Flu)	Flooding	Vacc Rate	Asthma Rate	Asthma Attack Rate	Pop Density	% Under 5	% Older 75	% Animal Production
**Avg Temp (Flood)**	1.00	0.49**	-0.52**	-0.51**	0.54**	0.25*	0.04	-0.30**	-0.04	-0.05	0.20*	0.31**	-0.33**	-0.27**
**Avg Temp (Flu)**	0.49**	1.00	0.15*	-0.24**	0.71**	0.90**	0.02	-0.29**	-0.34**	-0.34**	0.12	0.17*	-0.28**	-0.21*
**Relative Humidity (Flood)**	-0.52**	0.15*	1.00	0.38**	0.42**	0.32**	0.02	0.06	-0.16*	-0.17*	-0.19**	-0.22**	0.05	0.07
**Relative Humidity (Flu)**	-0.51**	-0.24**	0.38**	1.00	-0.23**	0.14*	0.03	0.17*	-0.14*	-0.13	-0.35**	-0.41**	0.34**	0.30**
**Absolute Humidity (Flood)**	0.54**	0.71**	0.42**	-0.23**	1.00	0.59**	0.05	-0.29**	-0.20**	-0.21**	0.03	0.12	-0.30**	-0.22**
**Absolute Humidity (Flu)**	0.25*	0.90**	0.32**	0.14*	0.59**	1.00	0.03	-0.24**	-0.43**	-0.43**	-0.01	-0.01	-0.17*	-0.12
**Flooding**	0.04	0.02	0.02	0.03	0.05	0.03	1.00	0.01	0.05	0.12	-0.17	-0.01	-0.14	0.07
**Vacc Rate**	-0.30**	-0.29**	0.06	0.17*	-0.29**	-0.24**	0.01	1.00	0.56**	0.47**	0.17*	-0.09	0.03	0.05
**Asthma Rate**	-0.04	-0.34**	-0.16*	-0.14*	-0.20**	-0.43**	0.05	0.56**	1.00	0.89**	0.28**	0.06	-0.21**	-0.28**
**Asthma Attack Rate**	-0.05	-0.34**	-0.17*	-0.13	-0.21**	-0.43**	0.12	0.47**	0.89**	1.00	0.33**	0.04	-0.08	-0.25**
**Pop Density**	0.20*	0.12	-0.19**	-0.35**	0.03	-0.01	-0.17	0.17*	0.28**	0.33**	1.00	0.59**	-0.50**	-0.71**
**% Under 5**	0.31**	0.17*	-0.22**	-0.41**	0.12	-0.01	-0.01	-0.09	0.06	0.04	0.59**	1.00	-0.53**	-0.57**
**%Older 75**	-0.33**	-0.28**	0.05	0.34**	-0.30**	-0.17*	-0.14	0.03	-0.21**	-0.08	-0.50**	-0.53**	1.00	0.82**
**%Animal Production**	-0.27**	-0.21*	0.07	0.30**	-0.22**	-0.12	0.07	0.05	-0.28**	-0.25**	-0.71**	-0.57**	0.82**	1.00

Avg: Average   Temp: Temperature   Vacc: Vaccination   *** p < 0.05**   **** p << 0.005**

Supplemental Table 6 Bayesian CAR model results using imputed environmental (set to 0) data and influenza diagnosis rates as the outcome

Variable	Mean	2.5% Quantile	97.5% Quantile	Gelman Diag.	Probability of risk > 1
Asthma Attack Rate	0.996	0.976	1.02	1.00	0.173
Vaccination Rate	1.00	1.00	1.00	1.00	≤0.001
Population Density	1.00	1.00	1.00	1.00	≤0.001
Percent in Animal Production	1.11	1.02	1.19	1.00	0.989
Average Absolute Humidity	1.56	0.889	2.77	1.00	0.941
Total Average Days Above Flood Stage	1.01	0.999	1.02	1.00	0.680

Supplemental Table 7 Bayesian CAR model results using imputed environmental (impute) data and influenza diagnosis rates as the outcome

Variable	Mean	2.5% Quantile	97.5% Quantile	Gelman Diag.	Probability of risk > 1
Asthma Attack Rate	0.996	0.976	1.02	1.00	0.186
Vaccination Rate	1.00	1.00	1.00	1.00	≤0.001
Population Density	1.00	1.00	1.00	1.00	≤0.001
Percent in Animal Production	1.11	1.02	1.20	1.00	0.992
Average Absolute Humidity	1.58	0.903	2.77	1.00	0.947
Total Average Days Above Flood Stage	1.01	0.999	1.02	1.00	0.740

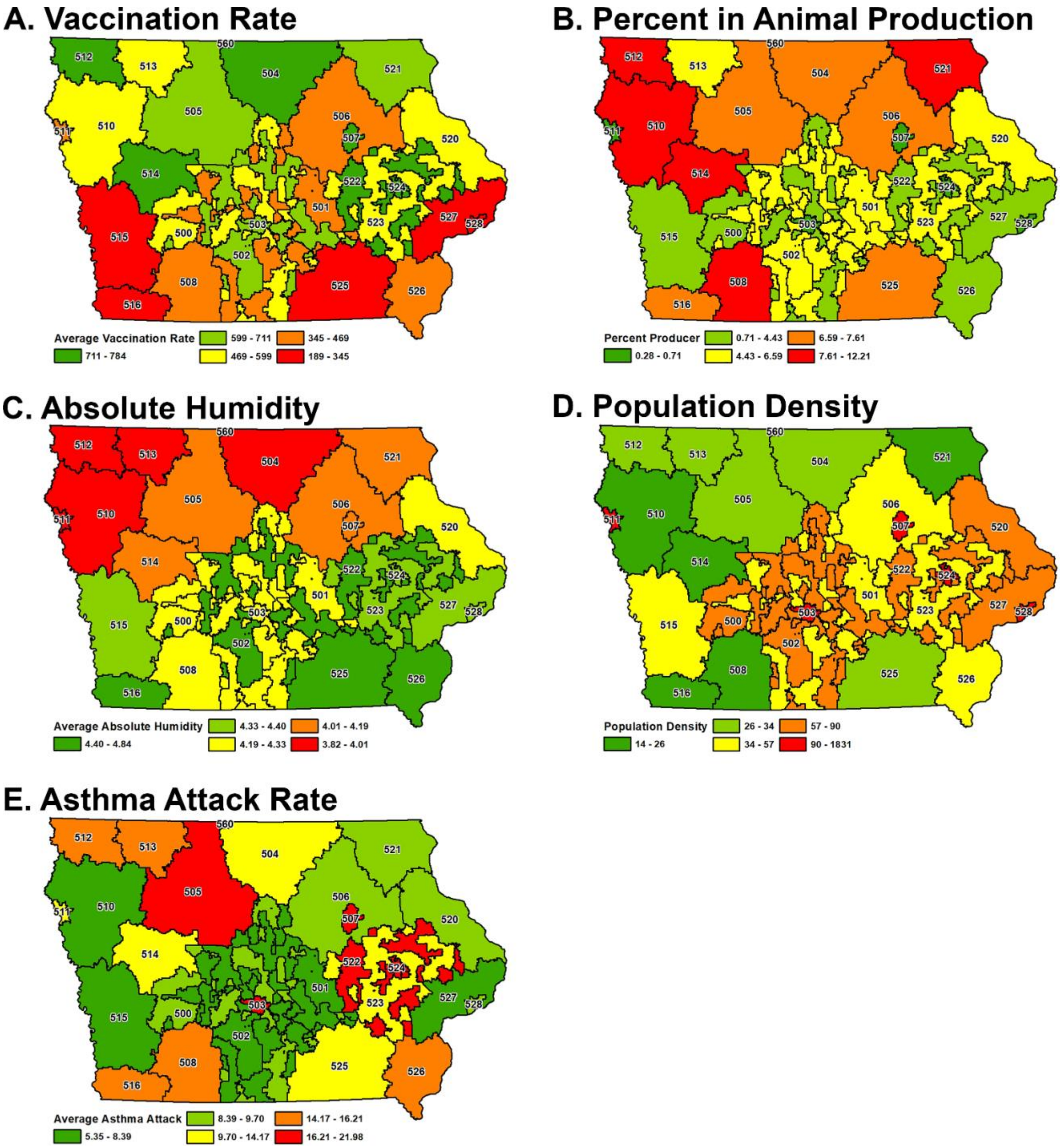

Supplemental Figure 1 Maps of other covariates used in modeling process. A) Vaccination Rates. Average vaccination per each three-digit ZCTA from 2007-2017. B) Percent of population working in Animal Production. Percent of each three-digit ZCTA total population that indicated Animal production as their primary vocation. C) Average Absolute Humidity. Average Absolute humidity from 2007-2017. D)Population Density. Population density for each three-digit ZCTA. E) Average Asthma Attack Rate. Average asthma attack rates per each three-digit ZCTA from 2007-2017.
